# Association of Vitamin D Receptor (VDR) Gene Polymorphisms with Osteoporotic Vertebral Fracture Risk: A Case–Control Study

**DOI:** 10.3390/genes17040410

**Published:** 2026-03-31

**Authors:** Nimetullah Alper Durmuş, Merdan Orunoglu, Sukru Oral, Rahmi Kemal Koç, Munis Dundar, Mehmet Meral

**Affiliations:** 1 Department of Neurosurgery, Faculty of Medicine, Erciyes University, 38030 Kayseri, Turkey; nimetullahalper@hotmail.com (N.A.D.); drsukruor@gmail.com (S.O.); kocrk@erciyes.edu.tr (R.K.K.); 2 Department of Neurosurgery, Kayseri State Hospital, 38010 Kayseri, Turkey; merdanorunoglu@gmail.com; 3 Department of Medical Genetics, Faculty of Medicine, Erciyes University, 38030 Kayseri, Turkey; mdundar@hotmail.com; 4 Department of Neurosurgery, Private Erciyes Hospital, 38020 Kayseri, Turkey

**Keywords:** osteoporosis, vertebral fracture, vitamin D receptor gene, VDR polymorphism, ApaI

## Abstract

Background/Objectives: Osteoporosis is a systemic skeletal disorder characterized by reduced bone mass and microarchitectural deterioration, resulting in an increased risk of fragility fractures, particularly vertebral fractures. Genetic factors are considered important in osteoporotic fracture susceptibility, and polymorphisms of the vitamin D receptor (VDR) gene have been widely studied because of their role in bone metabolism. To evaluate the distribution of VDR gene polymorphisms (FokI, BsmI, ApaI, and TaqI) in patients with osteoporotic vertebral fractures and to assess their association with fracture susceptibility. Methods: This case–control study included 86 individuals: 43 patients who underwent vertebroplasty for osteoporotic vertebral fractures and 43 osteoporotic individuals without vertebral fractures serving as controls. VDR gene polymorphisms ApaI, TaqI, BsmI and FokI were analyzed using real-time polymerase chain reaction. Genotype distributions were compared using Fisher’s exact test, and Hardy–Weinberg equilibrium was evaluated. Results: A significant difference between groups was observed only for the ApaI polymorphism (*p* = 0.002). The GG genotype was more frequent in patients, whereas the variant genotypes (GT and TT) were more prevalent in controls. The GG genotype was associated with an increased risk of vertebral fractures, while the presence of variant genotypes may be associated with reduced fracture susceptibility. No significant associations were found for TaqI, BsmI, or FokI polymorphisms. Conclusions: The ApaI polymorphism of the VDR gene may represent a protective genetic factor against osteoporotic vertebral fractures. In contrast, no associations were identified for the TaqI, BsmI, or FokI polymorphisms in this cohort. Larger studies in diverse populations are required to confirm these findings and to clarify the role of VDR gene variants in fracture susceptibility.

## 1. Introduction

Osteoporosis is a multifactorial and polygenic skeletal disorder characterized by a reduction in bone mass and deterioration of bone microarchitecture, leading to increased bone fragility and a higher risk of fractures. Although bone mineral density (BMD) is widely recognized as a primary determinant of osteoporotic fracture risk, it alone cannot fully account for fracture occurrence. Individuals with similar BMD values may exhibit markedly different fracture outcomes, suggesting that additional factors—particularly genetic determinants—may play a critical role in bone strength and fracture susceptibility independent of BMD. This discrepancy highlights the need to investigate genetic factors that may influence fracture susceptibility beyond bone mineral density. In addition to hormonal and environmental factors, genetic factors are also known to play an important role in the development of the disease [1]. Previous studies have demonstrated that genetic factors exert a significant influence on the pathogenesis of osteoporosis [2,3]. Furthermore, it has been suggested that genetic characteristics may account for approximately 50–85% of the variation observed in bone phenotype.

Polymorphism studies conducted within the scope of genetic research investigate the effects of various genes on disease development in different populations. Polymorphisms are defined as variations in DNA sequences that may lead to changes in gene function. Variations that occur at a frequency greater than 1% in a population are defined as polymorphisms, whereas those occurring at a frequency below 1% are referred to as mutations. The most common type of polymorphism in the human genome is the single-nucleotide polymorphism (SNP) [4,5]. SNPs play an important role in generating genetic diversity in the human genome and have been shown to contribute to the development of numerous diseases. These variations may influence physiological and biochemical pathways, thereby determining susceptibility to diseases and potentially altering responses to treatment [3,4]. In addition, genetic studies have shown that factors such as ethnicity, sex, age, and family history are important determinants influencing the effects of polymorphisms [6].

Numerous candidate genes associated with osteoporosis have been identified in studies aiming to elucidate the genetic basis of the disease. These include the vitamin D receptor gene (VDR), type I collagen alpha-1 chain gene (COL1A1), estrogen receptor gene (ESRα), interleukin receptor genes (IL-R), low-density lipoprotein gene (LDL), LDL receptor-related protein 5 (LRP5), androgen receptor gene (AR), progesterone receptor gene (PR), parathyroid hormone gene (PTH), and the parathyroid hormone receptor gene (PTHR1) [1,4,7].

The vitamin D receptor gene (VDR) is one of the key genes involved in the regulation of bone metabolism and calcium homeostasis. Vitamin D contributes to proper bone mineralization by regulating calcium and phosphate metabolism through its active hormonal metabolite, calcitriol [1,25(OH)_2_D_3_]. Vitamin D deficiency may lead to rickets in childhood and osteomalacia in adulthood, highlighting its important role in maintaining skeletal integrity [8]. In elderly individuals, vitamin D deficiency is more common due to reduced exposure to sunlight and decreased renal synthesis of vitamin D. Age-related vitamin D deficiency adversely affects calcium metabolism, leading to increased parathyroid hormone (PTH) levels, decreased bone mineral density, and an increased risk of fractures [9].

Vitamin D exerts its biological effects through the vitamin D receptor (VDR), a member of the steroid and thyroid hormone receptor family. Calcitriol binds to VDR as a ligand and regulates intestinal calcium absorption as well as renal reabsorption of calcium and phosphate. It also plays an important role in the regulation of parathyroid hormone levels. The VDR protein is one of the key regulatory molecules determining the effects of vitamin D on bone and calcium metabolism, exerting these effects by controlling the transcription of various genes [8].

The vitamin D receptor (VDR) gene, located on chromosome 12q13.11, consists of multiple exons encoding a nuclear receptor that regulates calcium homeostasis and bone metabolism. The gene structure allows the generation of different mRNA isoforms, and its expression is controlled by tissue-specific promoter regions [9].

Following binding to calcitriol, VDR forms a heterodimer with the retinoid X receptor (RXR), which translocates to the nucleus and binds to vitamin D response elements (VDREs) to regulate gene transcription. This process involves co-activator and co-repressor complexes that modulate chromatin structure and transcriptional activity [10].

Studies investigating VDR gene polymorphisms have reported heterogeneous results, potentially due to interactions with environmental factors such as vitamin D levels and calcium metabolism [11]. These polymorphisms have been suggested to influence fracture risk independently of bone mineral density and may alter responses to calcitriol therapy [12]. Although numerous studies have examined their effects on bone mineral density, findings remain inconsistent across different populations [13,14,15].

Several polymorphisms of the VDR gene have been identified. The FokI polymorphism, located in exon 2, affects the transcription initiation site and results in a shorter VDR protein, with potential effects on bone mineral density depending on demographic and clinical factors [16,17,18].

The BsmI polymorphism, located near intron 8–exon 9, is considered a silent variant but may influence gene expression through mRNA stability, with allele frequencies varying among populations [19,20].

The ApaI polymorphism arises from a single nucleotide substitution in intron 8, while the TaqI polymorphism results from a nucleotide change in exon 9 [14,21].

Vertebral fractures were specifically selected as the primary outcome because they are the most common fragility fractures associated with osteoporosis and are strongly related to increased morbidity and future fracture risk. In contrast to long bones, the vertebral bodies contain a higher proportion of trabecular (spongious) bone, which is more metabolically active and therefore more susceptible to osteoporotic changes. As a result, vertebral fractures may better reflect alterations in bone microarchitecture and quality beyond bone mineral density. Furthermore, vertebral fractures represent a clinically relevant and accessible patient population in spine-focused clinical practice, allowing for a more homogeneous and well-defined study cohort.

The aim of this study was to determine the presence and distribution of vitamin D receptor gene polymorphisms (FokI, BsmI, ApaI, and TaqI) in patients diagnosed with osteoporotic compression fractures and low bone mineral density (T-score < −2.5) and to compare these findings with the VDR gene polymorphisms of osteoporotic patients who have low bone mineral density but do not have vertebral fractures. Specifically, the primary aim was to compare the distribution of these polymorphisms between the fracture and non-fracture groups, while the secondary aim was to evaluate whether these polymorphisms may contribute to fracture susceptibility through biological mechanisms independent of bone mineral density and the bone remodeling cycle.

## 2. Materials and Methods

In this study, a total of 86 individuals were included, consisting of 43 patients who underwent vertebroplasty due to osteoporotic compression fractures between January 2021 and December 2024 and 43 osteoporotic individuals without fractures. Bone mineral density (BMD) values were measured in all participants. Individuals with a T-score higher than −2.5, as well as those with malignancy, rheumatologic diseases, or additional pathologies at the fracture site (such as tumors or infections), were excluded from the study.

The diagnosis of osteoporotic vertebral compression fractures was established based on clinical evaluation and radiological findings, including plain radiography and/or magnetic resonance imaging (MRI). High-energy trauma and secondary causes of vertebral fractures were excluded.

Written informed consent was obtained from all participants at the time of clinical procedures and was recorded in the hospital database. Participants were invited for evaluation, and blood samples were prospectively collected for genetic analysis. However, complete clinical data for all potential confounding variables (including age, body mass index, menopausal status, vitamin D levels, medication use, and comorbidities) were not available for all individuals. Therefore, detailed matching or multivariate adjustment was not performed.

The VDR gene polymorphisms investigated in this study were selected using the GWAS and ExAC databases along with a comprehensive literature review. Four VDR polymorphisms were determined for investigation: ApaI (rs7975232), TaqI (rs731236), FokI (rs10735810), and BsmI (rs1544410). The FokI polymorphism analyzed in this study (rs10735810) corresponds to rs2228570 in updated nomenclature.

Blood samples obtained from individuals included in the study were collected in 2 mL EDTA-containing blood tubes and stored at −80 °C until DNA isolation was performed. Genomic DNA was first isolated, after which the vitamin D receptor gene polymorphisms ApaI (rs7975232), TaqI (rs731236), FokI (rs10735810), and BsmI (rs1544410) were analyzed using real-time PCR.

### 2.1. Genomic DNA Isolation

Genomic DNA isolation from blood samples obtained from the volunteers was performed using the spin column method. This method enables rapid and efficient purification of nucleic acids using a solid-phase system. Nucleic acids bind to a solid phase consisting of a silica-based membrane. In the presence of chaotropic salts that disrupt cells and denature cellular proteins, DNA selectively binds to the silica membrane.

Genomic DNA isolation was carried out using a commercial kit (Zeesan Blood DNA Extraction Kit, Catalog No: 25047701, Zeesan, Xiamen, China). After the necessary preparation of the components included in the kit, the isolation procedure was performed according to the manufacturer’s protocol using the Zeesan Nucleic Acid Extraction System (Zeesan Lab-Aid824s, 501101, Xiamen, China).

### 2.2. Fluorescence Melting Curve Analysis

**Figure 1:**
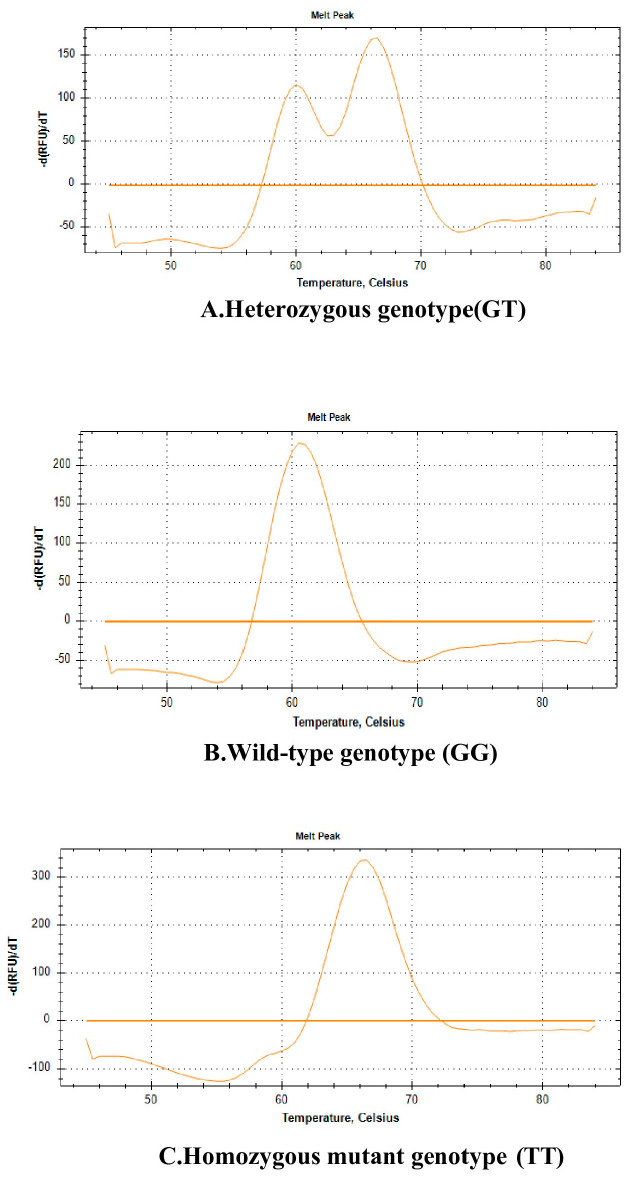
Fluorescence melting curve analysis results for the ApaI rs7975232 (G > T) polymorphism. Different curves represent distinct genotypes: (**A**) Heterozygous genotype (GT), (**B**) wild-type genotype (GG), (**C**) homozygous variant genotype (TT).

The determination of VDR gene polymorphisms (rs7975232 ApaI, rs731236 TaqI, rs1544410 BsmI, and rs10735810 FokI) was performed using the Fluorescence Melting Curve Analysis (FMCA) method. This method involves the analysis of melting curves of DNA regions amplified by polymerase chain reaction (PCR). Target regions were amplified using specific primers, and genotypes were determined through analysis of the melting curves (Figure 1).

### 2.3. Real-Time Polymerase Chain Reaction (Real-Time PCR)

In the real-time PCR analysis, the FMCA method was used to detect single-nucleotide polymorphisms rs7975232 ApaI (G > T), rs731236 TaqI (T > C), rs1544410 BsmI (G > A), and rs10735810 FokI (T > C). SNP-brand Vitamin D Receptor Real-Time PCR kits (Catalog No: 29R-10-04) were used for this purpose.

Using specifically designed primer–probe sets, a reaction mixture with a total volume of 25 µL was prepared for each well. Allele-specific master mixes provided within the kit were used for each rs variant. The reaction mixture consisted of 5 µL of DNA sample and 20 µL of Master Mix, which included 4 µL of forward primer (2 mM concentration), 2 µL of reverse primer (20 mM concentration), and 2 µL of probe (20 mM concentration), supplied ready-to-use within the kit.

For negative control purposes, distilled water was added to one of the wells. The prepared reaction mixtures were sealed carefully with transparent caps to prevent contamination. Subsequently, all wells were placed into a PCR device (Bio-Rad CFX96, (Bio-Rad Laboratories, Hercules, CA, USA)) to perform the reaction and obtain readings under appropriate RT-PCR conditions.

### 2.4. Statistical Analysis

All statistical analyses were performed using SPSS software (Statistical Package for the Social Sciences), version 22.0 (IBM Corp., Armonk, NY, USA). Categorical variables were expressed as numbers (*n* and column percentages (%).

Fisher’s exact test was used to compare genotype distributions of vitamin D receptor (VDR) gene polymorphisms between the patient and control groups due to the presence of low expected cell frequencies. The same statistical method was also applied to compare genotype distributions according to sex.

The conformity of genotype distributions with Hardy–Weinberg equilibrium (HWE) was evaluated in both the patient and control groups using the chi-square goodness-of-fit test by comparing observed and expected genotype frequencies.

Odds ratios (ORs) and 95% confidence intervals (CIs) were calculated to estimate the strength of association between genotypes and fracture risk. In all statistical analyses, a *p*-value of <0.05 was considered statistically significant. To account for multiple comparisons, the Bonferroni correction was applied, and the adjusted level of statistical significance was set at *p* < 0.0125. The null hypothesis of this study was that there is no statistically significant difference in the distribution of VDR gene polymorphisms between osteoporotic patients with and without vertebral fractures.

This study was approved by the local ethics committee and conducted in accordance with the principles of the Declaration of Helsinki.

## 3. Results

A total of 86 individuals were included in the study. Of these, 43 patients constituted the osteoporotic vertebral fracture group, while the remaining 43 individuals formed the control group. Among all participants, 48 were female (55.8%) and 38 were male (44.2%).

Genotype and allele frequencies of vitamin D receptor (VDR) gene polymorphisms, including ApaI (rs7975232), TaqI (rs731236), BsmI (rs1544410), and FokI (rs10735810) in the patient group are presented in Table 1. For the ApaI (rs7975232) polymorphism, the genotype distribution in the patient group was GG 32.6% (*n* = 14), GT 44.2% (*n* = 19), and TT 23.2% (*n* = 10). The allele frequencies for this locus were calculated as 54.7% for the G allele and 45.3% for the T allele. For the TaqI (rs731236) polymorphism, genotype frequencies in the patient group were TT 51.2% (*n* = 22), TC 46.5% (*n* = 20), and CC 2.3% (*n* = 1), with allele frequencies of 74.4% for the T allele and 25.6% for the C allele. In the case of the BsmI (rs1544410) polymorphism, genotype distributions were GG 41.9% (*n* = 18), GA 48.8% (*n* = 21), and AA 9.3% (*n* = 4), with allele frequencies of 66.3% for the G allele and 33.7% for the A allele. For the FokI (rs10735810) polymorphism, genotype distributions in the patient group were TT 2.3% (*n* = 1), TC 41.9% (*n* = 18), and CC 55.8% (*n* = 24), with allele frequencies of 23.3% for the T allele and 76.7% for the C allele (Table 1).

Genotype and allele frequencies of VDR gene polymorphisms in the control group are shown in Table 2. For the ApaI polymorphism, genotype distributions in the control group were GG 4.7% (*n* = 2), GT 53.5% (*n* = 23), and TT 41.9% (*n* = 18), with allele frequencies of 31.4% for the G allele and 68.6% for the T allele. For the TaqI polymorphism, genotype frequencies in the control group were TT 41.9% (*n* = 18), TC 53.5% (*n* = 23), and CC 4.7% (*n* = 2). For the BsmI polymorphism, genotype distributions were GG 37.2% (*n* = 16), GA 48.8% (*n* = 21), and AA 14.0% (*n* = 6). Regarding the FokI polymorphism, genotype frequencies were TT 9.3% (*n* = 4), TC 37.2% (*n* = 16), and CC 53.5% (*n* = 23) in the control group (Table 2).

A comparison of genotype distributions of VDR gene polymorphisms between the patient and control groups is presented in Table 3. A statistically significant difference was observed only for the ApaI (rs7975232) polymorphism between the two groups (*p* = 0.002). The GG genotype was more frequent in the patient group, whereas the TT genotype was more frequent in the control group. The GG genotype was associated with an increased risk of vertebral fractures (OR ≈ 9.9), while the presence of variant genotypes was associated with reduced fracture susceptibility. This association remained statistically significant after Bonferroni correction. In contrast, no statistically significant differences were detected between the patient and control groups for the TaqI (*p* = 0.630), BsmI (*p* = 0.790), or FokI (*p* = 0.528) polymorphisms (Table 3).

Genotype distributions of VDR gene polymorphisms were also evaluated according to sex, and the results are presented in Table 4. The analyses revealed no statistically significant differences in genotype distributions between female and male patients for any of the investigated polymorphisms, including ApaI (*p* = 0.744), TaqI (*p* = 0.580), BsmI (*p* = 0.434), and FokI (*p* = 1.000). These findings indicate that the distribution of VDR gene polymorphisms was independent of sex in the study population (Table 4).

The conformity of genotype distributions with Hardy–Weinberg equilibrium (HWE) was also evaluated, and the results are presented in Table 3 and Table 4. The analyses demonstrated that genotype distributions for all investigated polymorphisms were consistent with the Hardy–Weinberg equilibrium in both the patient and control groups. The HWE *p*-values for the ApaI (rs7975232) polymorphism were *p* = 0.477 in the patient group and *p* = 0.113 in the control group. For the TaqI (rs731236) polymorphism, the HWE *p*-values were *p* = 0.201 in the patient group and *p* = 0.712 in the control group. For the BsmI (rs1544410) polymorphism, the HWE *p*-values were *p* = 0.439 in the patient group and *p* = 0.712 in the control group. Finally, for the FokI (rs10735810) polymorphism, the HWE *p*-values were *p* = 0.257 in the patient group and *p* = 0.622 in the control group. Since all *p*-values were greater than 0.05, the observed genotype distributions were considered to be consistent with the Hardy–Weinberg equilibrium. No deviation from the Hardy–Weinberg equilibrium was detected for any of the studied polymorphisms.

## 4. Discussion

Osteoporosis is a multifactorial disease characterized by decreased bone mineral density and deterioration of bone microarchitecture, leading to an increased risk of fractures and significant morbidity, particularly due to vertebral fractures. Although bone mineral density (BMD) is one of the primary determinants of osteoporotic fracture development, recent studies have demonstrated that genetic factors also play an important role in determining fracture susceptibility [1,3,5]. Among the candidate genetic variations associated with bone metabolism and fracture risk, polymorphisms in the vitamin D receptor (VDR) gene have received considerable attention [11,15].

In the present study, a statistically significant difference between the patient and control groups was observed for the ApaI (rs7975232) polymorphism. Notably, the wild-type GG genotype was more frequently observed in patients with osteoporotic vertebral fractures, whereas the variant genotypes (GT and TT) were more prevalent in the control group.

Importantly, further analysis demonstrated that individuals carrying the GG genotype had a markedly increased risk of vertebral fractures (OR = 9.90; 95% CI: 2.09–46.91), indicating a strong association between this genotype and fracture susceptibility. In contrast, the presence of variant genotypes was associated with reduced fracture susceptibility. This finding provides clinically meaningful evidence supporting the role of VDR gene polymorphisms in fracture risk beyond simple genotype distribution comparisons.

Although the ApaI polymorphism is located in a non-coding region of the VDR gene, it may influence gene expression through mechanisms such as mRNA stability and transcriptional regulation. Previous studies have demonstrated that VDR polymorphisms may affect calcium absorption, bone turnover markers, and bone microarchitecture. In particular, alterations in VDR activity have been associated with changes in osteoblast and osteoclast function, which are key determinants of bone quality.

Importantly, fracture risk is not solely determined by bone mineral density but is also influenced by bone quality, including microarchitecture and material properties. Therefore, the observed association in this study may reflect differences in bone quality rather than bone mineral density alone. However, given the lack of adjustment for potential confounding factors, this relationship should be interpreted with caution.

Taken together, these findings suggest that the ApaI polymorphism may be associated with fracture susceptibility through biological mechanisms not fully explained by BMD. Nevertheless, this association does not imply direct clinical applicability, and further large-scale, prospective, and multi-center studies are required to validate these findings and to determine whether VDR genotyping could contribute to risk stratification in clinical practice. Although the ApaI polymorphism is located in a non-coding region of the VDR gene, it may influence gene expression by affecting mRNA stability or transcriptional regulation [22]. Previous studies have suggested that VDR polymorphisms may alter calcium absorption, bone turnover markers, and bone microarchitecture. In particular, variations in VDR activity have been associated with changes in bone remodeling dynamics, including osteoblast and osteoclast function, which are critical determinants of bone quality [23,24].

Importantly, fracture risk is not solely dependent on bone mineral density but is also influenced by bone quality, including microarchitecture and material properties. Therefore, the observed association in our study may reflect differences in bone quality rather than bone mineral density alone. These findings support the hypothesis that ApaI polymorphism may contribute to fracture susceptibility through biological mechanisms independent of BMD, potentially explaining its apparent protective association in the control group.

In a cohort study conducted by Garnero et al., the influence of vitamin D receptor (VDR) gene polymorphisms on osteoporotic fracture risk in postmenopausal women was investigated. In this study, four major VDR gene variants—BsmI, TaqI, ApaI, and FokI—were examined. The authors reported that women carrying specific alleles of the BsmI, ApaI, and TaqI polymorphisms exhibited a higher risk of osteoporotic fractures, whereas the FokI polymorphism was not associated with fracture risk. Importantly, this association was found to be independent of bone mineral density. In other words, even in individuals without markedly reduced BMD, the presence of certain genetic variants may increase the likelihood of fractures. These findings suggest that the VDR gene may influence bone quality and microarchitecture through mechanisms independent of BMD. Furthermore, the study highlighted that specific polymorphisms in the VDR gene may serve as important genetic markers for predicting fracture risk in postmenopausal women. These results emphasize that, in the evaluation of osteoporosis, not only bone mineral density but also genetic factors should be taken into consideration [25]. In contrast to these findings, the higher frequency of polymorphic genotypes observed in the control group in our study suggests that ApaI variants may be associated with reduced fracture susceptibility; however, this finding should be interpreted with caution.

In another study involving 100 postmenopausal Turkish women, the BsmI (B/b) polymorphism in the VDR gene and the Sp1 (S/s) polymorphism in the COL1A1 gene were examined. The results demonstrated no statistically significant association between the VDR BsmI polymorphism or the COL1A1 Sp1 polymorphism and either low BMD or vertebral fractures. Thus, in this Turkish population, VDR polymorphisms were not found to be associated with fracture occurrence or reduced BMD [26]. Consistent with these findings, our study did not demonstrate a significant difference between the patient and control groups in terms of the BsmI polymorphism. This observation suggests that the effects of VDR polymorphisms may vary across different populations.

In a meta-analysis conducted by Shen et al., the effect of VDR gene polymorphisms on fracture risk in postmenopausal women was evaluated. By combining data from multiple studies, the authors focused particularly on common VDR gene variants, including BsmI, FokI, ApaI, and TaqI. Their findings indicated that VDR gene polymorphisms did not show a consistent or significant association with fracture risk. Consequently, these genetic variants were not considered strong clinical predictors of fracture risk in postmenopausal women. The study emphasized that although the VDR gene plays an important role in bone metabolism, the currently identified genetic variations are insufficient for predicting fracture risk [27]. Similarly, in our study, no significant association was observed for the TaqI, BsmI, and FokI polymorphisms.

In a meta-analysis conducted by Mu et al. in 2022, a total of 23 studies involving 5844 osteoporotic fracture cases and 19,339 controls were analyzed. The polymorphisms evaluated included BsmI, ApaI, TaqI, FokI, and Cdx2. Initial analyses suggested an increased fracture risk associated with the ApaI polymorphism in European populations and the BsmI polymorphism in American populations [28]. However, after excluding low-quality studies and those not in Hardy–Weinberg equilibrium, the credibility of these positive findings was considered low. Overall, the study concluded that there was no significant association between VDR polymorphisms (BsmI, ApaI, TaqI, FokI, and Cdx2) and osteoporotic fracture risk [28].

Horst-Sikorska et al. conducted a study in 2013 investigating the relationship between BsmI, ApaI, and TaqI polymorphisms in the vitamin D receptor gene and bone mineral density as well as fracture risk in postmenopausal women with osteoporosis. A total of 501 women from Poland were included in the study, and BMD measurements along with vertebral and non-vertebral fracture histories were evaluated. No significant association was found between VDR genotypes and BMD, and the genotypes did not significantly affect overall fracture risk. However, women carrying certain alleles (ApaI “a”, BsmI “b”, and TaqI “T”) exhibited a higher risk of non-vertebral fractures. These findings suggest that VDR polymorphisms may influence fracture risk through mechanisms independent of BMD [29]. In our study, the significant association observed for the ApaI polymorphism suggests that this polymorphic variant may play a protective role against the development of osteoporotic vertebral fractures. However, no significant association was observed for the other VDR polymorphisms examined, which is consistent with the heterogeneous findings reported in the literature.

In a study conducted by Mondockova et al., five VDR gene polymorphisms (ApaI, TaqI, BsmI, FokI, and Cdx2) were investigated. The study included 356 postmenopausal women from Slovakia and evaluated the relationships among bone mineral density, biochemical parameters, bone turnover markers, fracture prevalence, and responses to antiresorptive therapies (estrogen–progesterone therapy, raloxifene, and ibandronate) [30].

The BsmI polymorphism was found to be significantly associated with lumbar spine BMD as well as serum osteocalcin (OC) and β-CrossLaps levels, which are markers of bone resorption. ApaI and Cdx2 polymorphisms were associated with bone turnover markers such as osteocalcin and alkaline phosphatase, whereas the TaqI polymorphism was associated with all bone turnover markers. Furthermore, ApaI, TaqI, and BsmI genotypes increased the risk of spinal, radial, or total fractures by approximately 2.03- to 3.17-fold.

Overall, all treatment modalities (estrogen–progesterone therapy, raloxifene, and ibandronate) improved bone density and biochemical parameters. However, genetic variations influenced treatment responses, particularly to ibandronate and raloxifene therapy. For example, women carrying the ApaI/aa, TaqI/TT, and BsmI/bb genotypes exhibited weaker or absent responses to ibandronate treatment in terms of BMD improvement. In raloxifene therapy, increases in lumbar spine BMD were associated particularly with TaqI and BsmI polymorphisms. Consequently, this study demonstrated that VDR gene polymorphisms may influence not only bone mineral density and biochemical bone turnover markers but also fracture risk and responses to anti-osteoporotic therapies. These findings highlight the potential importance of personalized treatment strategies based on genetic profiles [30]. In our study, although treatment response was not directly assessed, the significant association observed between the ApaI polymorphism and fracture susceptibility further supports the potential role of VDR genetic variations in influencing bone fragility and clinical outcomes. These findings suggest that ApaI genotyping may have potential value in identifying individuals at increased risk of osteoporotic vertebral fractures and could contribute to future screening strategies. However, further large-scale and prospective studies are required before clinical implementation can be recommended.

This study has several limitations. First, the sample size of the study population was relatively small. Second, the study was conducted using a single-center design. Nevertheless, the fact that genotype distributions in both the patient and control groups were consistent with the Hardy–Weinberg equilibrium supports the reliability of the genetic analyses performed in this study.

## 5. Conclusions

In this study, the results of the analysis indicated a significant association for the ApaI polymorphism between the patient and control groups. The higher frequency of the polymorphic genotypes in the control group suggests that ApaI variants may be associated with reduced susceptibility to the development of osteoporotic vertebral fractures. In contrast, no significant associations were identified for the TaqI, BsmI, and FokI polymorphisms. These findings indicate that the ApaI polymorphism may represent a potentially protective genetic factor in osteoporotic vertebral fracture susceptibility. However, further studies involving larger and more diverse populations are needed to confirm and better clarify this relationship.

## Figures and Tables

**Table 1:** Distribution of genotype and allele frequencies of VDR gene polymorphisms in patients with osteoporotic vertebral fractures and the control group, along with Hardy–Weinberg equilibrium analysis.

Polymorphism (rs no.)	Group	Genotype Distribution (*n*)	Genotype Frequency (%)	Allele Frequency	HWE *p*-Value
rs7975232 (ApaI) G > T	Patients (*n* = 43)	GG:14/GT:19/TT:10	32.6/44.2/23.2	G:54.7%/T:45.3%	0.477
	Controls (*n* = 43)	GG:2/GT:23/TT:18	4.7/53.5/41.9	G:31.4%/T:68.6%	0.113
rs731236 (TaqI) T > C	Patients (*n* = 43)	TT:22/TC:20/CC:1	51.2/46.5/2.3	T:74.4%/C:25.6%	0.201
	Controls (*n* = 43)	TT:18/TC:23/CC:2	41.9/53.5/4.7	T:68.6%/C:31.4%	0.712
rs1544410 (BsmI) G > A	Patients (*n* = 43)	GG:18/GA:21/AA:4	41.9/48.8/9.3	G:66.3%/A:33.7%	0.439
	Controls (*n* = 43)	GG:16/GA:21/AA:6	37.2/48.8/14.0	G:61.6%/A:38.4%	0.712
rs10735810 (FokI) T > C	Patients (*n* = 43)	TT:1/TC:18/CC:24	2.3/41.9/55.8	T:23.3%/C:76.7%	0.257
	Controls (*n* = 43)	TT:4/TC:16/CC:23	9.3/37.2/53.5	T:27.9%/C:72.1%	0.622

Data are presented as genotype counts (*n*) and percentages (%). Allele frequencies represent the total frequency of the alleles for each polymorphism. Hardy–Weinberg equilibrium (HWE) was assessed using the chi-square goodness-of-fit test by comparing observed and expected genotype frequencies. A *p*-value > 0.05 indicates that genotype distributions are consistent with the Hardy–Weinberg equilibrium. HWE: Hardy–Weinberg equilibrium.

**Table 2:** Comparison of observed genotype frequencies and expected genotype frequencies according to Hardy–Weinberg equilibrium in the patient and control groups.

Polymorphism (rs no.)	Group	Observed Genotypes	Expected Genotypes	HWE *p*-Value
rs7975232 (ApaI)	Patients (*n* = 43)	14/19/10	12.84/21.31/8.84	0.477
	Controls (*n* = 43)	2/23/18	4.24/18.52/20.24	0.113
rs731236 (TaqI)	Patients (*n* = 43)	22/20/1	23.77/16.47/2.76	0.201
	Controls (*n* = 43)	18/23/2	20.28/18.47/4.24	0.712
rs1544410 (BsmI)	Patients (*n* = 43)	18/21/4	18.94/18.10/5.96	0.439
	Controls (*n* = 43)	16/21/6	16.35/20.36/6.29	0.712
rs10735810 (FokI)	Patients (*n* = 43)	1/18/24	2.33/15.35/25.33	0.257
	Controls (*n* = 43)	4/16/23	3.35/17.30/22.35	0.622

Observed genotype values represent the actual genotype counts obtained in the study, whereas expected genotype values represent the theoretical genotype frequencies calculated under the Hardy–Weinberg equilibrium assumption. Hardy–Weinberg equilibrium analysis was performed using the chi-square goodness-of-fit test. A *p*-value > 0.05 indicates that genotype distribution is consistent with the Hardy–Weinberg equilibrium.

**Table 3:** Comparison of genotype distributions of VDR gene polymorphisms between the osteoporotic vertebral fracture patient group and the control group.

Polymorphism (rs no.)	Genotype	Patients (*n* = 43)	%	Controls (*n* = 43)	%	*p*
rs7975232 (ApaI)	GG	14	32.6	2	4.7	**0.002**
	GT	19	44.2	23	53.5	
	TT	10	23.3	18	41.9	
rs731236 (TaqI)	TT	22	51.2	18	41.9	0.630
	TC	20	46.5	23	53.5	
	CC	1	2.3	2	4.7	
rs1544410 (BsmI)	GG	18	41.9	16	37.2	0.790
	GA	21	48.8	21	48.8	
	AA	4	9.3	6	14.0	
rs10735810 (FokI)	TT	1	2.3	4	9.3	0.528
	TC	18	41.9	16	37.2	
	CC	24	55.8	23	53.5	

Data are presented as number (*n*) and column percentage (%). Fisher’s exact test was used to compare genotype distributions between the patient and control groups. A *p*-value < 0.05 was considered statistically significant.

**Table 4:** Comparison of genotype distributions of VDR gene polymorphisms according to sex.

Polymorphism (rs no.)	Genotype	Female (*n* = 48)	%	Male (*n* = 38)	%	*p*
rs7975232 (ApaI)	GG	8	16.7	8	21.1	0.744
	GT	25	52.1	17	44.7	
	TT	15	31.3	13	34.2	
rs731236 (TaqI)	TT	20	41.7	20	52.6	0.580
	TC	26	54.2	17	44.7	
	CC	2	4.2	1	2.6	
rs1544410 (BsmI)	GG	16	33.3	18	47.4	0.434
	GA	26	54.2	16	42.1	
	AA	6	12.5	4	10.5	
rs10735810 (FokI)	TT	3	6.3	2	5.3	1.000
	TC	19	39.6	15	39.5	
	CC	26	54.2	21	55.3	

Data are presented as number (*n*) and column percentage (%). Fisher’s exact test was used to compare genotype distributions according to sex. A *p*-value < 0.05 was considered the threshold for statistical significance.

## Data Availability

The datasets analyzed during the current study are available from the corresponding author upon reasonable request.

## Abbreviations

VDR	Vitamin D Receptor
SNP	Single Nucleotide Polymorphism
BMD	Bone Mineral Density
PCR	Polymerase Chain Reaction
Real-Time PCR	Real-Time Polymerase Chain Reaction
EDTA	Ethylenediaminetetraacetic Acid
SPSS	Statistical Package for the Social Sciences
HWE	Hardy–Weinberg Equilibrium
